# Pressure-Promoted Triplet-Pair Separation in Singlet-Fission TIPS-Pentacene Nanofilms Revealed by Ultrafast Spectroscopy

**DOI:** 10.3390/nano14181487

**Published:** 2024-09-13

**Authors:** Lu Wang, Ruixue Zhu, Ruihua Pu, Weimin Liu, Yang Lu, Tsu-Chieu Weng

**Affiliations:** 1 School of Physical Science and Technology, Shanghai Tech University, Shanghai 201210, China; 2 Center for Transformative Science, Shanghai Tech University, Shanghai 201210, China; 3 Center for High Pressure Science & Technology Advanced Research, Shanghai 201203, China; 4 Shanghai Key Laboratory of Material Frontiers Research in Extreme Environments (MFree), Shanghai Advanced Research in Physical Sciences (SHARPS), Shanghai 201203, China

**Keywords:** singlet fission, amorphous films, diamond anvil cell, transient absorption

## Abstract

Singlet fission (SF), as an effective way to break through the Shockley–Queisser limit, can dramatically improve energy conversion efficiency in solar cell areas. The formation, separation, and relaxation of triplet-pair excitons directly affect the triplet yield, especially triplet-pair separation; thus, how to enhance the triplet-pair separation rate becomes one of the key points to improve SF efficiency; the decay mechanism where the singlet state is converted into two triplet states is significant for the study of the SF mechanism. Herein, we employ ultrafast transient absorption spectroscopy to study the singlet-fission process of nano-amorphous 6, 13-bis(triisopropylsilylethynyl)-Pentacene (TIPS-pentacene) films in a diamond anvil cell (DAC). A kinetics model related to the structural geometric details, as well as an evaluation of the pressure manipulation impacts, is demonstrated based on the experimental results. The results indicate that pressure manipulation enhanced the triplet-pair separation rates of SF-based materials according to their structural micro-environmental improvement when compressed in DAC, while the triplet-exciton transportation lifetime is prolonged. This work shows that pressure may effectively optimize the structural disorder of SF materials, which were found to improve triplet-pair separation efficiency and potentially offer an effective way to further improve SF efficiency.

## 1. Introduction

Singlet fission (SF) is a photophysical process that can produce two triplet excitons from a single absorbed photon, which is a spin-allowed process that can be viewed as a radiationless transition of internal conversion [1]. This characteristic can be utilized to enhance solar cell efficiency beyond the Shockley–Queisser limit, thus attracting strong scientific attention to raise the quantum efficiency of the solar cells field with the SF process [2]. When the molecule is excited by light, it will transition from the ground state $S_{0}$ to the singlet $S_{1}$. Then the excited molecule S_1_ will transfer energy to the adjacent molecules in the ground state $S_{0}$, resulting in two triplet molecules $T_{1}$, which is shown in Equation (1). Ehrler et al. [3] successfully demonstrated the use of pentacene nanocrystals as SF sensitizers for increasing the efficiency of amorphous silicon solar cells. For SF sensitizers to be used in practice, they need to fulfill many conditions, including 200% triplet yield, long triplet lifetime, strong visible absorption from 2.2 eV, redox potential, and other properties conducive to effective charge separation, and long-term stability under light [4].

(1) $$S_{1}S_{0}⇌T_{1}+T_{1},$$

Pentacene and its derivatives have long triplet lifetimes and efficient fission rates, and they have been targeted as future SF materials and photoelectric materials. As the paradigm, pentacene and its derivatives are currently the most researched and intensively studied materials in the SF field [5]. Such efficient systems are models and targets for future SF sensitizers and disordered devices that generate excited states from sunlight. The SF process of pentacene is exothermic, so the SF process can occur quickly and efficiently. By the substitution of triisopropyl silicon-acetylene (TIPS) into pentaphene 6, 13 positions, 6, 13-bis triisopropyl silicon-acetylene (TPN) can be obtained, which can improve the poor stability and poor solubility of the deficiency effectively, and thus is more straightforward in preparing thin films used in actual optoelectronic devices [6]. 

In order to increase intermolecular coupling and thus increase singlet-fission efficiency, some researchers have attempted to adjust the property of intermolecular coupling and investigate its effect on SF. Methods of changing the intermolecular coupling that have been explored include designing covalently bound dimers [7,8], group substitution [9,10], crystallizing singlet fissile materials into polycrystalline forms [11,12], and co-crystallizing SF sensitizers with inert materials to increase intermolecular spacing [13,14]. All the above studies have demonstrated that the kinetics of triplet state transfer is sensitive to intermolecular coupling, and accelerating the separation of triplet pairs is an effective way to harvest multiple triplets more efficiently from SF [15].

The amorphous structure is proven to suppress the relaxation processes in the pentacene derivatives during the singlet-fission processes [16]. TPN films exhibit an ultrafast SF process depending on the morphology of the film (<100 fs) [12,17,18,19]. Grieco et al. reported that the intermolecular order of TPN films and crystal structure could be systematically adjusted by solvent annealing crystallization treatment [19]. Munson et al. used ultrafast infrared spectroscopy to detect the relevant triplet states after singlet fission in amorphous and crystalline pentaphenyl films [12]. The results indicated that triplet diffusion in amorphous films is an order of magnitude lower than that in crystalline analogs, that triplet trap states limit the transport of triplet excitons in amorphous films, and that the various intermolecular couplings, which are caused by the different crystal structure of thin films, affect triplet separation and transfer dramatically. Many researchers have studied the different SF characteristics of amorphous versus crystalline structures of TPN; transversing the crystallization into polymorphs or amorphous structures can tune intermolecular coupling, which can redistribute the electron density and energy levels of the SF sensitizer, and finally affect triplet-pair separation. There is no consensus yet on which structure is more conducive to improving the efficiency of the SF process. Therefore, it is essential to figure out how intermolecular coupling affects the separation of triplet pairs and the transmission process [15].

Compared with traditional chemical technology, high-pressure technology is a non-invasive physical way of regulating intermolecular coupling. Moderate pressure will decrease the molecular distance and cause changes in the intensity of non-radiative vibrations, resulting in changes in the excited states [20]. According to this pressure-induced energy-level change, the singlet-fission process can be modulated efficiently. Doucette et al. [21] investigated the solid-state pressure effect of TPN films by using the DAC technology, revealing an effective triplet-pair separation. Kinoshita et al. [22] used hydrostatic pressure to modulate the dimeric solution of p-pentacene, revealing hydrostatic pressure-induced formation and dissociation of the associated triplet pairs in SF. 

Herein, we investigate a prototypical singlet-fission system of amorphous 6, 13-bis(triisopropylsilylethynyl)pentacene (TPN) films with nano-scale thickness. Pressure manipulation according to a diamond anvil cell (DAC) was used to tune the intermolecular coupling of TPN films, whose appropriate intermolecular coupling forces enable high-efficiency triplet-pair separation instead of fast internal conversion deactivation pathways. Ultrafast transient absorption spectroscopy was employed to probe the related triplet-pair dynamics and excited state lifetime; moreover, correlated triplet-pair separation enhanced according to the pressure manipulation was also investigated. Exponential function fitting is performed to test the kinetic models and elucidate the relaxation pathways probed in transient absorption experiments. The ultrafast transient absorption results show that the introduction of moderate structural disorder through exerting pressure can indeed act to enhance the SF process, which is indicated by the promoted dissociation of triplet pairs and the increased ratio of free-triplet states; this study gives a potential new way of modifying the structure of candidate SF materials by pressure manipulation to optimize triplet exciton generation.

## 2. Materials and Methods

### 2.1. Materials and Preparation

All commercial reagents and solvents were used as received without further purification. 6, 13-bis(triisopropylsilylethynyl) pentacene (97%) was obtained from Adamas-beta. Amorphous TPN films were prepared via spin coating method. 

Place the Sapphire substrate with a thickness of 50 μm in a glass cleaner solution and soak it at 50 °C for 1 h, then clean it with an ultrasonic cleaner for 20 min, rinse with deionized water, clean it with an ultrasonic cleaner for 20 min, and then wash it in ethanol for 30 min.

**Figure 1:**
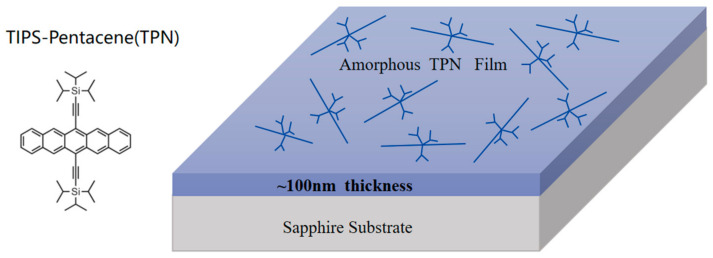
The chemical formula of TPN and the nano-amorphous thin film with sapphire as substrate.

The CH_2_Cl_2_ solution of TPN (concentration 20 mg/mL) was stirred for 8 h and then spin-coated on the cleaned glass substrate at a speed of 30 s/3000 rpm to obtain the blue films, as shown in Supporting Information Figure S1. These films were amorphous TPN thin films with a thickness of approximately 100 nm (Figure 1) [11,12].

### 2.2. Experiential Instrumentation

#### 2.2.1. High-Pressure Device: Diamond Anvil Cell

**Figure 2:**
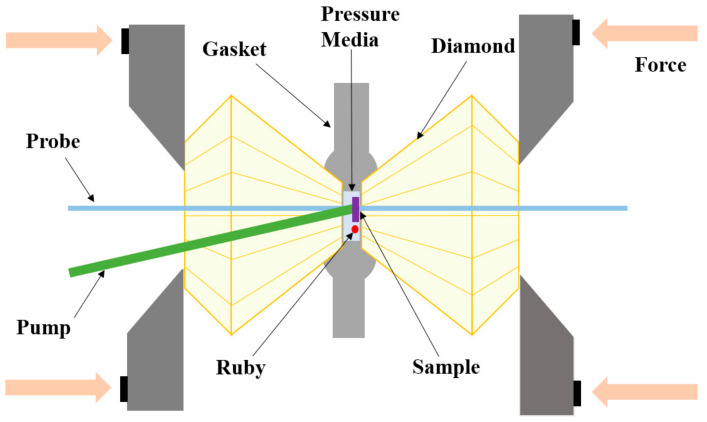
The structure diagram of the Diamond Anvil Cell.

The TPN films prepared on sapphire glass with a thickness of 50 μm were used as the starting material. The films were loaded into a Mao–Bell-type symmetric diamond anvil cell (DAC, 800 μm culets, Figure 2). T301 stainless steel foil was pre-indented and a hole was laser drilled at the indentation center to serve as the gasket and sample chamber. Silicone oil was used as the pressure-transmitting medium (PTM) and the pressure was calibrated by measuring the frequency shift of the ruby R1 fluorescence line [23]. Please note that silicone oil can provide a hydrostatic environment at a pressure below ~6 GPa [24].

#### 2.2.2. Femtosecond Time-Resolved Transient Absorption System

Transient absorption (TA) was carried out on a commercial transient absorption microscope (Time-Tech Spectra, Dalian, China). The 1030 nm fiber laser was generated from a commercial Fiber laser (~205 fs, 100 kHz repetition rate, CARBIDE, Light Conversion Inc, Vilnius, Lithuania). A BBO crystal was employed for the second harmonic generation of the 1030 nm wavelength to generate an excitation pump at 515 nm. A chopper operating at a frequency of 500 Hz was positioned in the pump beam path. For this study, the probe pulse employed a white light continuum spanning approximately from ~400 nm to ~960 nm, generated by a sapphire plate with a thickness of 2 mm. The diamond anvil can be placed at the intersection of the pump light and the probe light to perform transient absorption measurements under pressure [25].

## 3. Results and Discussion

### 3.1. Steady-State Spectra in Amorphous TPN Films under High-Pressure

We used a new high-pressure loading method. Firstly, the amorphous thin films of TPN were prepared on the surface of our ultra-thin sapphire by spin coating, and then the sapphire fragments coated with sample films were used for high-pressure loading, as shown in Figure S2 in the Supporting Information. 

**Figure 3:**
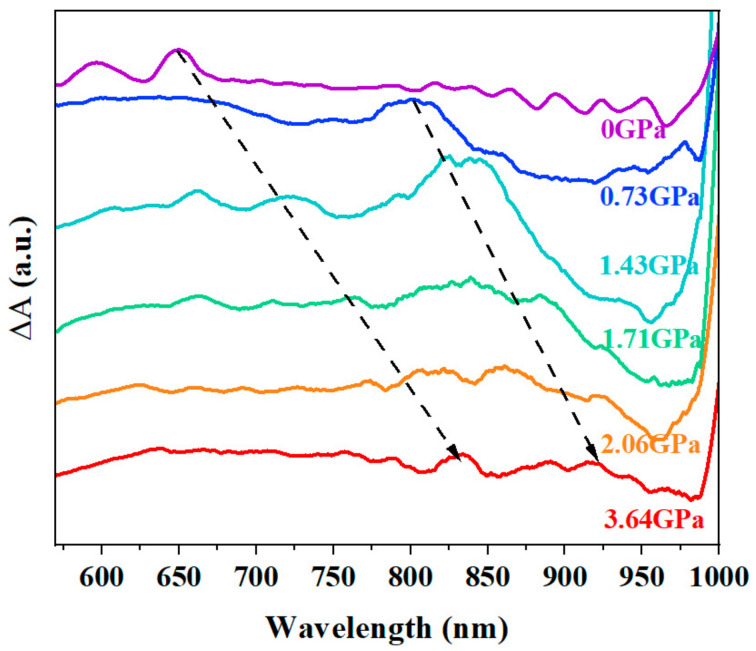
Pressure-dependent steady-state absorption spectra of amorphous TPN film.

The high-pressure steady-state spectral characterization of amorphous thin film was conducted under 3.64 GPa. The steady-state absorption spectra at different pressures are shown in Figure 3, and more details are provided in the Supporting Information, Figure S3. The Raman signal also moves toward a higher wavenumber as the pressure increases [26], as shown in the Supporting Information, Figure S4. 

Figure 3 represents the micro-Vis–NIR absorption spectra of the amorphous film of TPN that was measured during compression in the DAC over a range of pressures from atmospheric to 3.64 GPa. The absorption spectrum of the amorphous TPN film at atmospheric pressure (0.0 GPa) has two absorption peaks at around 600 and 650 nm, which are characteristic of the transition within the S_0_→S_1_ [11]. At elevated pressures, the positions of the absorption peaks shift, indicating changes in the energies of the electronic states under the applied pressure. With the increase in pressure, the molecular spacing gradually decreases, the morphology of the films changes, and the energy gap is decreased, which causes the positions of steady-state absorption peaks to shift to longer wavelengths gradually.

### 3.2. Transient Absorption Spectra in Amorphous TPN Films under High Pressure

The SF pathways in TPN films remain a subject of ongoing debate [27,28]. In TPN films, the singlet-fission process is more complex than that shown in Equation (1). The SF process described in Equation (2) is widely accepted [29]. In this process, excitons in the excited state S_1_ are coupled to neighboring molecules in the ground state S_0_, resulting in bound-triplet pairs ^1^(TT). The bound-triplet pair ^1^(TT) is entangled into an overall singlet and is an essential intermediate for the formation of two free-triplet excitons, where the correlated triplet ^1^(T···T).^1^(T…T) represents the triplet pair that has lost electronic coupling but retains spin coherence, and is also equivalent to the singlet spin multiplicity of a whole. The triplet pair subsequently separates into two individual triplets (T_1_) as they move away from each other [30].

(2) $$S_{1}S_{0}{⇌}^{1}(TT){⇌}^{1}(T\cdots T)⇌T_{1}+T_{1},$$

**Figure 4:**
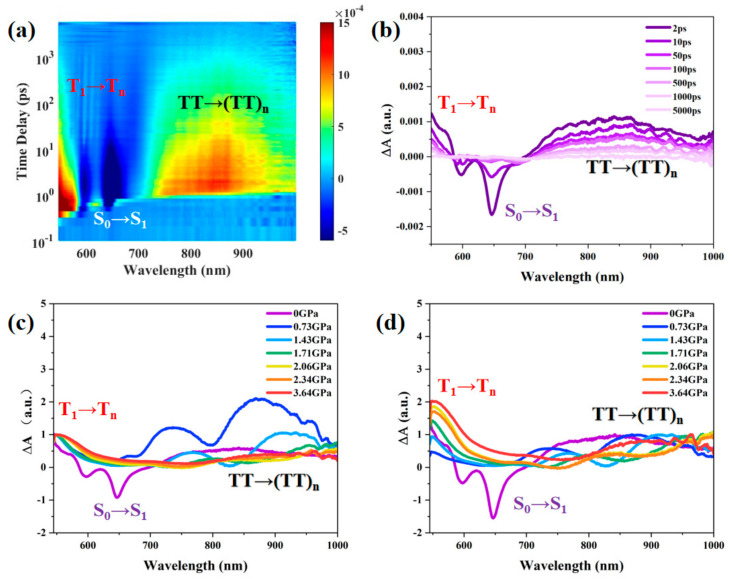
(**a**,**b**) Femtosecond transient absorption spectra of TPN amorphous film under atmospheric pressure with 515 nm excitation. (**c**,**d**) Comparison of the fs-transient absorption spectra of TPN amorphous film under various pressures. Normalizing the characteristic peaks at free triplets and the pairs of bound triplets, it can be observed that the characteristic peaks of both free-triplet and bound-triplet pairs move toward the long wave with the increase in pressure.

In order to further investigate, the characterization of transient absorption under high pressure was carried out to study the effect of pressure on singlet fission. Using a femtosecond time-resolved transient absorption spectroscopy system, the kinetic process of singlet fission in TPN amorphous films was characterized using 515 nm excitation light as the pump light. We measured high-pressure transient absorption using a commercial fiber laser (~205 fs, 100 kHz, CARBIDE, Light Conversion Inc.). A BBO crystal was employed for second harmonic generation at a wavelength of 1030 nm, resulting in excitation pump light at 515 nm with a chopper frequency of 500 Hz. During the high-pressure transient absorption tests, we excited the sample inside the press using pump light at 100 kHz with a pulse energy of 35 μJ, and the probe light ranged from 550 nm to 1000 nm. The experimental results of the same TPN amorphous film under different pressures are shown in Figure 4. In addition, to ensure the reliability of the data, we repeated the experiment and obtained consistent results.

We examined the influence that changes in intermolecular coupling during compression in the DAC have on the transition energies and lifetime of triplet excitons that form in TPN amorphous films following singlet fission. Figure 4 illustrates the pump and probe beam geometries in the DAC used for the fs-transient absorption measurements and transitions of the free-triplet T_1_ and triplet-pair exciton (TT)_1_. The transient absorption spectra below the diagram were measured at a 2 ps time delay following optical excitation at 515 nm and 35 mJ/cm^2^ of TPN amorphous films under a range of pressures from atmospheric to 3.6 GPa. More details of transient absorption at other pressures are presented in the Supporting Information in Figure S5. The TA data reveal a pronounced shift to longer wavelengths of the T_1_/T_n_ transition with increasing pressure.

Three spectral features are apparent in the transient absorption spectra, [31,32,33,34]. The two negative-going bleaches at 600 nm and 650 nm correspond to the amorphous films bleach of S_0_/S_1_ absorptions, as shown in Figure 4a,b. These spectral characteristics are attributed to the absorption originating from excited states with different multiplicities The excited state absorption appears around 550~600 nm assigned to the free-triplets T_1_/T_n_ transition [19,32,34]. The near-infrared excited state absorption band, spanning the spectral range from 700 nm to greater than 1000 nm, represents the transition of triplet pairs (TT)_1_/(TT)_n_ [31,33]. The excited absorption peaks of the free triplets are different from the bound-triplet pairs, so it is easy to distinguish them. Therefore, we observe the changes in free-triplets and bound-triplet pairs under pressure according to the intensity and lifetime of characteristic peaks at different wavelengths. 

As shown in Figure 4c,d, after normalizing the characteristic peaks of triplet pairs and free triplets, we can observe that with the increase in pressure, the spectra of the ground state bleaching peak and the excited state absorption characteristic peak of the ground state are gradually red-shifted, and the full width at half maximum of the characteristic peak gradually widens. The position of the ground-state bleaching peak under different pressures is consistent with that of the steady-state absorption peak shown in Figure 3. For example, under a pressure of 0.73 GPa, the steady-state absorption peak red-shifts from 650 nm under normal pressure to 800 nm, and at the same time, the characteristic peak of ground-state bleaching also moves to 800 nm. As pressure increases, the bonding of the C-C stretching mode is disrupted, leading to an increase in the dipole moment of the triplet excited state. Due to the increase in effective conjugation length, the electronic structure of the triplet state becomes delocalized. Aromatic C-C stretching vibrations can produce strong coupling between two conjugated main chains, thereby affecting triplet–triplet energy transitions. Consequently, the peak position of the TT transition rapidly shifts to longer wavelengths.

**Figure 5:**
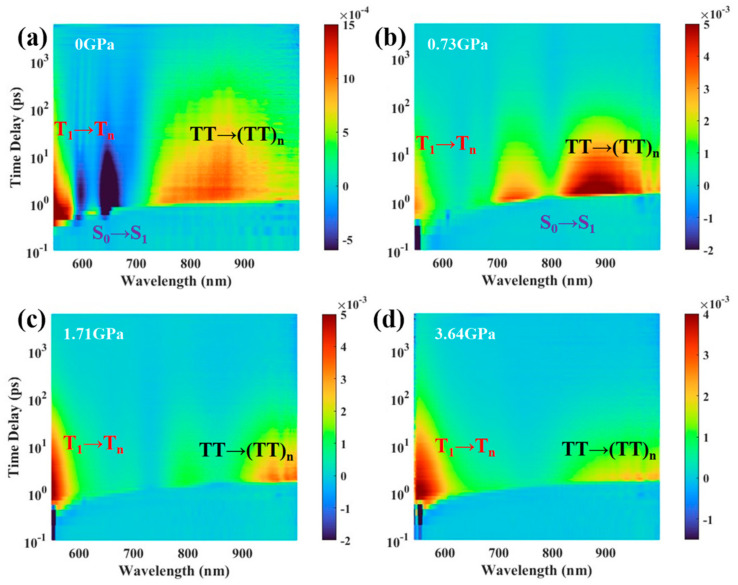
Comparison of the fs-transient absorption spectra of TPN amorphous film under (**a**) 0 GPa, (**b**) 0.73 GPa, (**c**) 1.71 GPa, and (**d**) 3.64 GPa pressures, which shows that the proportion of free triplets and bound triplets changes with the increase in pressure.

To conduct a more rigorous comparison of the proportional representation and lifetime variations of triplet pairs and free triplets across various pressures, we employ a comprehensive two-dimensional wavelength–time–amplitude overview to further analyze the transient absorption spectrum of amorphous TPN films. For amorphous TPN films, applying pressure can effectively reduce the intermolecular distance. This compression increases the overlap of π planes, thereby enhancing π–π interactions. This improved interaction causes a red shift in the absorption spectrum and increases the proportion of triplet excitons. These changes are directly attributed to the impact of pressure on molecular packing. It can be observed that during the pressurization process of 0~0.73 GPa, the ground-state bleaching peak of the singlet state gradually decreases, and the proportion of bound-triplet pairs gradually increases (Figure 5). In this pressure range, the intensification of intermolecular coupling promotes the formation of bound-triplet pairs (TT)_1_ by singlet fission, and the quantum yield of the triplet state in the first step of singlet fission increases. During the pressurization process from 0.73 GPa to 2.64 GPa, the proportion of bound-triplet pairs decreased gradually, and the proportion of free-triplet states increased gradually, indicating that the split into free-triplet pairs mainly occurred under this period of pressure, which improved the yield of singlet fission. The singlet state significantly affects pressure at a pressure of 0.73 GPa, and the triplet state is more sensitive to pressure above 0.73 GPa.

We define the overall SF quantum yield [35] as $\phi_{SF}$:

(3) $$\phi_{SF}=\frac{max([T_{1}])}{max([S_{1}])},$$

while the yield including the first SF step (S_1_ + S_0_ → ^1^(TT)) $\phi_{SF}^{\prime }$ is given by

(4) $$\phi_{\mathrm{SF}}^{\prime }=\frac{max([T_{1}]+2\times {[}^{1}(TT)])}{max([S_{1}])},$$

The experimental results showed that pressure can significantly promote the efficiency of singlet fission and the yield of triplet states. The amorphous thin films had a high degree of disorder, promoting the formation of triplet pairs in singlet fission before 0.73 GPa. From 0.73 GPa to 3.84 GPa, pressure promoted the dissociation of triplet pairs, significantly increasing the proportion of triplet states, so that the ratio of triplet pairs and free-triplet states can be adjusted through pressure regulation.

**Figure 6:**
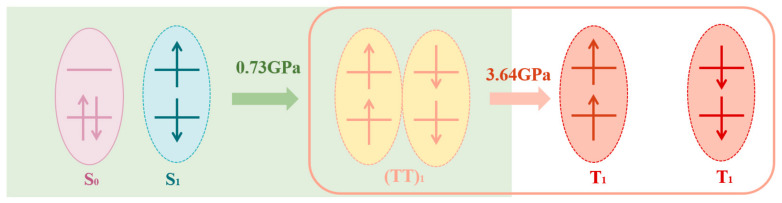
Diagram of singlet-fission process facilitated by different pressures. Pressure under 0.73 GPa promotes singlet fission into triplet pairs, then pressure between 0.73 GPa and 3.64 GPa promotes triplet pairs into free triplets.

**Figure 7:**
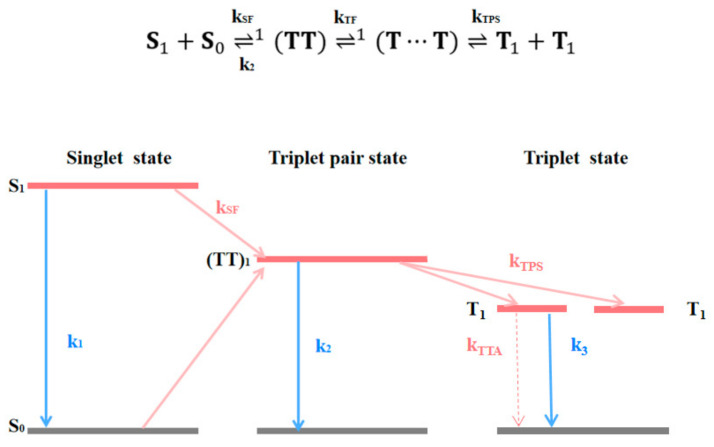
The schematic diagram of the effect of pressurization on the promotion of singlet fission in amorphous TPN films.

**Figure 8:**
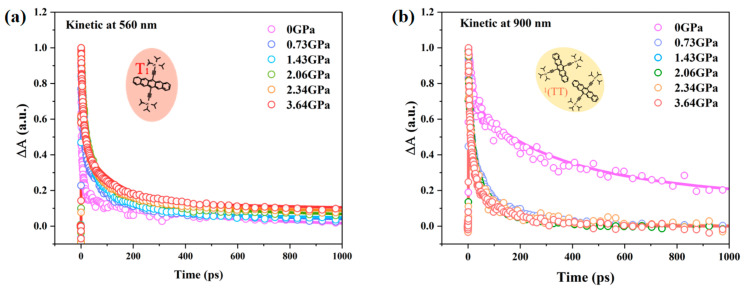
Comparison of the dynamics of TPN amorphous film under various pressures: (**a**) The kinetics of free triplets at 560 nm, and (**b**) the kinetics of triplet pairs at 900 nm. The points in the figure represent the collected experimental data points, while the curve represents the data fitting of the constructed model.

Based on the above data and analysis, we have obtained a complete schematic diagram of pressure-promoted singlet fission, as shown in Figure 6. Under low pressure, the process that is mainly promoted is the fission of the singlet state into triplet pairs, while under higher pressure, the promoted process is the separation of triplet pairs into free triplets in the previous fission process. We used a tri-exponential dynamics model shown in Figure 7 to obtain ultrafast rise and relaxation times at various probe wavelengths. The kinetic analysis was performed by comparing the TA kinetics at two characteristic probe wavelengths at 560 nm, and 900 nm, as shown in Figure 8. The fitted lifetime data are shown in Tables S1 and S2 in the Supporting Information. The range of emission signals under pressure is between 600 and 740 nm, as shown in Figure S6 in the Supporting Information, which does not significantly impact the kinetics.

(5) $$\frac{d[S_{1}S_{0}]}{dt}=-k_{SF}[S_{1}S_{0}],$$

(6) $$\frac{d{[}^{1}(TT)]}{dt}=+k_{SF}[S_{1}S_{0}]-k_{TPS}{[}^{1}(TT)]+k_{TF}{[}^{1}(T\ldots T)],$$

(7) $$\frac{d{[}^{1}(T\ldots T)]}{dt}=+k_{TPS}{[}^{1}(TT)]-k_{TF}{[}^{1}(T\ldots T)]-\frac{1}{2}k_{ST}{[}^{1}(T\ldots T),$$

(8) $$\frac{d[T_{1}]}{dt}=+2k_{ST}{[}^{1}(T\ldots T)]-\frac{1}{2}k_{TTA}[T_{1}{]}^{2},$$

(9) $$\tau =\frac{1}{k},$$

In this kinetic model, $k_{SF}$ is the primary singlet-fission rate to form the ^1^(TT) state that occurs on the ∼100 fs timescale, $k_{TPS}$ is the rate of triplet-pair separation to create the ^1^(T…T) sate that occurs on the few picosecond timescale, $k_{TF}$ is the rate of fusion to reform the ^1^(TT) state from the ^1^(T…T) state, $k_{ST}$ is the rate of irreversible separation of the ^1^(T…T) state, and $k_{TTA}$ is the decay coefficient describing triplet–triplet annihilation (TTA). At the same time, k_1_ represents the rate at which the singlet relaxes back to the ground state, while k_2_ is related to triplet pairs, and k_3_ represents the free-triplet rate.

The kinetics of free triplets at 560 nm shows that the lifetime of free triplet T_1_ increases with the increase in pressure, which directly indicates that pressure increases the relaxation time of the free triplet. The result is contrary to the dynamics of crystal films [21], as shown in Figure S7 in the Supporting Information. This may be due to the increased pressure that promotes intermolecular disorder and inhibits the mobility of triplet states. However, the (TT)_1_ decay at 900 nm is less regular than that of T_1_. This observation suggests that, besides the TTA process, other competitive interactions may exist between the two constituent triplet excitons. Nanofilm morphology and pressure could differently influence these interactions, thus exhibiting varying dependencies. Amorphous films do not have long-range ordered crystal structures as compared to crystalline films, as shown in the Supporting Information, Figure S8. External forces alter the molecular packing pattern of amorphous TPN films, increasing disorder [35,36]. This increased disorder hinders the relaxation process of free-triplet excitons, leading to extended relaxation lifetimes [37,38,39,40]. Therefore, by modulating the molecular packing of amorphous TPN films through external forces, effective control over triplet exciton dynamics can be achieved. It also demonstrates that during the SF process, when the intermolecular coupling strength is too weak, the SF process finds it challenging to compete with other deactivation pathways, resulting in inefficient SF. Increasing the pressure within a specific range to promote intermolecular coupling can enhance the yield of singlet-fission triplets and shorten the relaxation lifetime of triplet pairs, facilitating their separation into free triplets. However, the intermolecular coupling strength should not be too strong, as excessive coupling is not conducive to the formation of independent triplets. Therefore, to optimize the speed and efficiency of the SF process, fine adjustments must be made to the molecular stacking configuration to achieve the optimal intermolecular coupling strength.

## 4. Conclusions

In summary, we studied the singlet-fission reaction mechanisms of amorphous TPN films under various pressure values by femtosecond transient absorption spectroscopy, DAC was used as a pressure manipulation device to study the effect of pressure on the singlet-fission process. This work has demonstrated that the singlet-fission reaction dynamics of amorphous TPN films depend significantly upon the pressure values, and the enhanced structural disorder of amorphous TPN films under pressure manipulation indicated that the presence of this disorder might be responsible for the enhanced SF rates that we observed. Physical pressurization via the DAC increased the disorder among amorphous film molecules while simultaneously enhancing the coupling between these disordered molecules. Ultrafast transient absorption results indicate substantial evidence in the dynamics of both faster triplet-pair separations and a longer triplet-exciton lifetime for the SF process that evolves under pressure, and improved rates of SF for amorphous TPN films under pressure can be identified. The results showed that physical pressurization increased the coupling between disordered molecules in amorphous thin films. This facilitated the generation of bound-triplet pairs up to 0.73 GPa and promoted the dissociation of triplet pairs between 0.73 GPa and 2.34 GPa, resulting in a significant increase in free triplets. Additionally, the extended relaxation time of these augmented free triplets back to the ground state suggests a prolonged carrier lifetime. The lifetime of the triplet is extended from 400 ps at atmospheric pressure to nanoseconds, which holds significant implications for the development of solar cells.

## Data Availability

The data presented in this study is contained within this article.

## Supplementary Materials

The following supporting information can be downloaded at: https://www.mdpi.com/article/10.3390/nano14181487/s1, Figure S1: Photographs of the amorphous TPN films fragments; Figure S2: The amorphous TPN film (blue) and fully crystalline (gray) film; Figure S3: Steady-state absorption spectra of TPN films under different pressures: (a) crystalline TPN films, (b) amorphous TPN films; Figure S4: Raman signal of TPN film under pressure, (532 nm excitation); Figure S5: Transient absorption spectra of amorphous TPN film at (a) 0 GPa, (b) 0.73 GPa, (c) 1.71 GPa, (d) 2.06 GPa, (e) 2.34 GPa, (f) 3.64 GPa; Figure S6: Steady-state fluorescence spectra of TPN films under different pressures (532 nm excitation); Figure S7: Transient dynamics of TPN crystalline films under various pressure values; Figure S8: XRD characterization of amorphous TPN films versus solvent-annealed TPN crystalline films; Table S1: Amorphous TPN film—Free-triplets kinetic fitting at 560 nm; Table S2: Amorphous TPN film—Triple-state bondage pairs kinetic fitting at 900 nm.
