# Supplementary material for: Pressure-Promoted Triplet-Pair Separation in Singlet-Fission TIPS-Pentacene Nanofilms Revealed by Ultrafast Spectroscopy
Source: Nanomaterials (Basel). 2024 Sep 13;14(18):1487. doi: 10.3390/nano14181487 (PMC11434892; doi:10.3390/nano14181487)
Supplement: Supplementary file 1 [file nanomaterials-14-01487-s001.zip › nanomaterials-3086273-supplementary.pdf]

Supporting Information

Pressure-Promoted Triplet-Pair Separation in Singlet-Fission TIPS-Pentacene Nanofilms Revealed by Ultrafast Spectroscopy

Lu Wang[1], Ruixue Zhu[2,*], Ruihua Pu[1], Weimin Liu[1], Yang Lu[3,4,*], Tsu-Chieu Weng[1,2,*]

[1] School of Physical Science and Technology, Shanghai Tech University, Shanghai 201210, China;
[2] Center for Transformative Science, Shanghai Tech University, Shanghai 201210, China;
[3] Center for High Pressure Science & Technology Advanced Research, Shanghai 201203, China;
[4] Shanghai Key Laboratory of Material Frontiers Research in Extreme Environments (MFree), Shanghai Advanced Research in Physical Sciences (SHARPS), Shanghai 201203, China;

* Correspondence: zhurx@shanghaitech.edu.cn (R.Z.); yang.lu@hpstar.ac.cn (Y.L.); wengzq@shanghaitech.edu.cn (T.C.W.)

Figures:

Amorphous TPN film prepared by spin coating and it can be annealed by solvent vapor (SVA) to obtain crystalline TPN films[1].

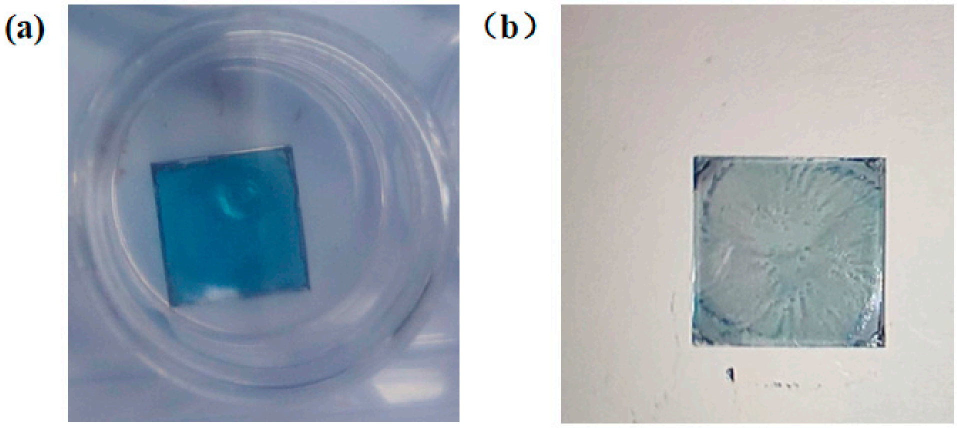

Figure S1 The amorphous TPN Amorphous TPN film (blue) and fully crystalline (gray) film

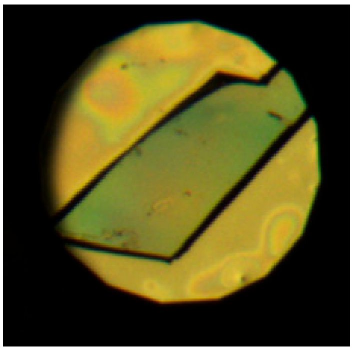

Figure S2 Photographs of the amorphous TPN film fragments

We conducted pressure-dependent steady-state absorption measurements on both crystalline and amorphous forms of TPN, as compared in Figure S3. At 0 GPa, amorphous TPN films exhibit only two absorption peaks within the tested range, at

approximately 600-700 nm. However, upon 0.73 GPa, a characteristic peak emerges at 800 nm, which is quite similar to the absorption peak of the crystalline film under pressure, so we speculate part of the crystal structure is produced during the pressurization of the amorphous film. Besides, the redshift trend indicated by dotted arrows is due to the decreased energy gap as the pressure increases.

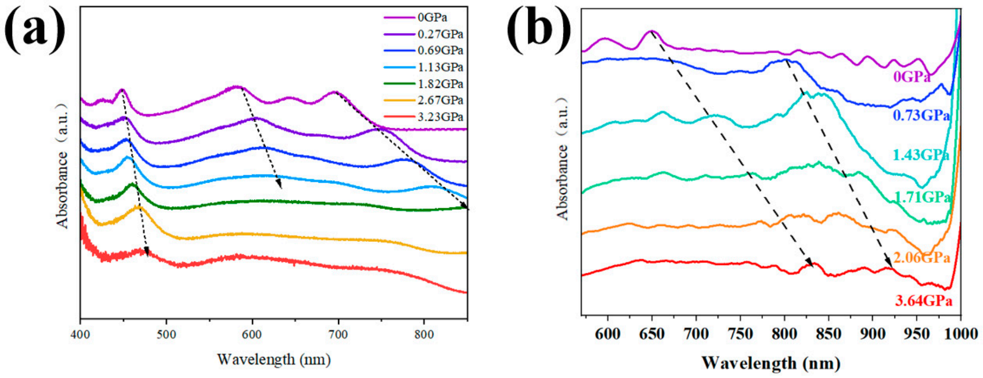

Figure S3 Steady-state absorption spectra of TPN films under different pressures (a) crystalline TPN films (b) amorphous TPN films

The Raman signal at 1332 cm^{-1} is the Raman signal of diamond, which moves towards a higher wavenumber as the pressure increases. The peak at 1157 cm^{-1} at atmospheric pressure corresponds to the C-H bending vibration at the end of the aromatic ring of TPN, and 1373 cm^{-1} corresponds to the expansion and contraction vibration of the C-C ring in the TPN backbone, mainly along the minor axis of the molecule. 1577cm^{-1} corresponds to the telescopic vibrations of C-C along the elongated molecular axis, respectively. When the pressure increases, the characteristic peaks of the TPN film move to a high wavenumber.

Raman spectroscopy can reveal the vibrational characteristics of molecules under compression. Mulazzi et al. calculated the Raman spectra of organic materials with different conjugation lengths under pressure, and the results showed that the electronic states with longer conjugation lengths have stronger interactions with the side chain modes[2]. Pressure studies on Raman spectra have indicated that TPN exhibits higher

structural planarity under higher pressures[3], facilitating easier electron transport along the backbone and resulting in electron delocalization.

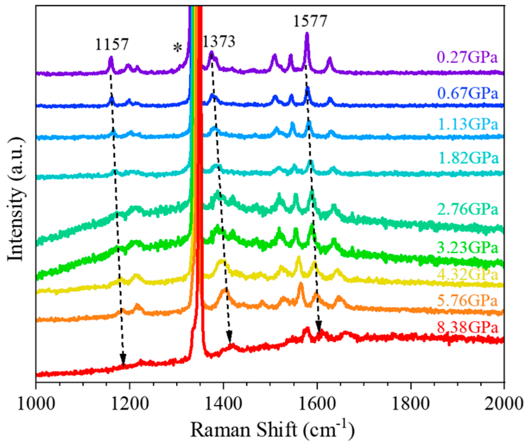

Figure S4 Raman signal of TPN film under pressure (532 nm excitation).

During the high-pressure transient absorption tests, we excited the sample inside the press using pump light at 100 kHz with a pulse energy of 35 μJ, and the probe light ranged from 550 nm to 1000 nm.

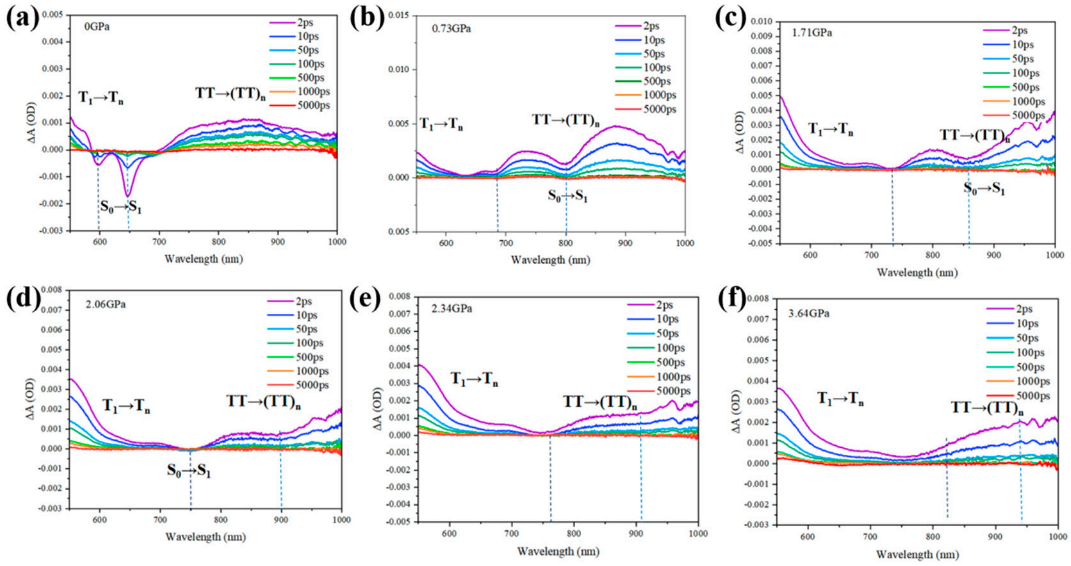

Figure S5 Transient absorption spectra of amorphous TPN film at (a)0GPa(b)0.73GPa(c)1.71GPa(d)2.06GPa(e)2.34GPa(f)3.64 GPa.

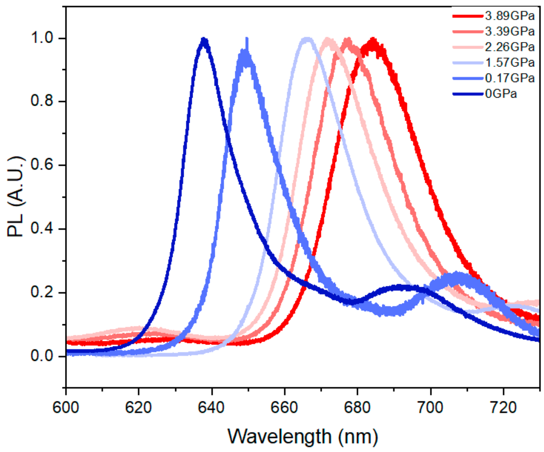

Figure S6 Steady-state fluorescence spectra of TPN films under different pressures (532 nm excitation)

We have measured the high-pressure steady-state emission spectra of TPN, and observed a red-shift in the emission peaks with increasing pressure, within the range of 600-740 nm, which does not significantly impact the kinetics at 560 nm and 900 nm.

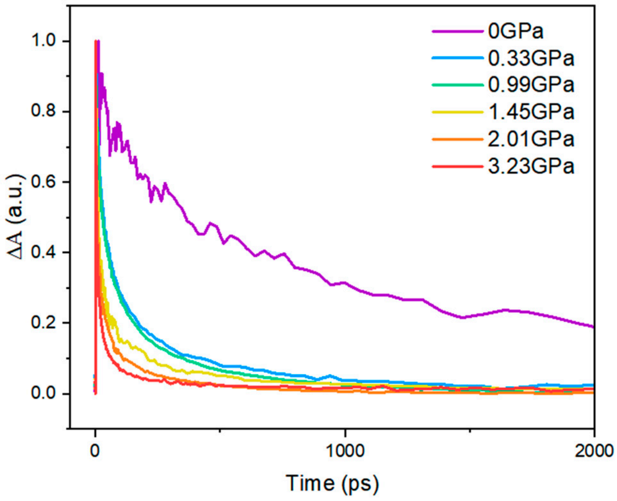

Figure S7 Transient dynamics of TPN crystalline films under various pressure values

In order to better explain the changes in singlet fission process of amorphous TPN films after pressurization, we also made a comparison of the transient absorption of TPN crystal films under the same experimental conditions. The kinetic results show that the lifetime of the triplets in crystalline films decreases with the increase of pressure.

However, the triplet lifetime of amorphous film relaxation back to the ground state is prolonged, which is contrary to the results of crystal films.

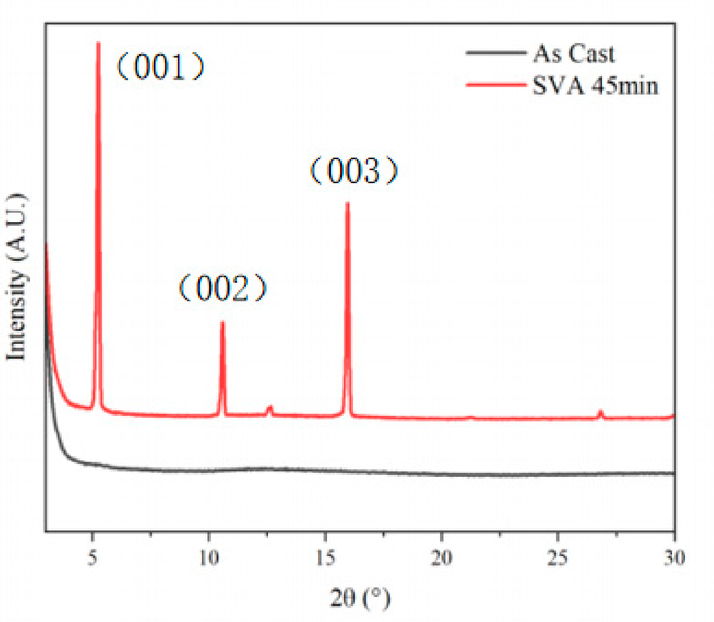

Figure S8 XRD characterization of amorphous TPN films (black curve) versus solvent-annealed TPN crystalline films (red curve)

Tables:

Kinetic analysis was performed using a tri-exponential fitting approach at 560 nm and 900nm, Specific data are shown in the following table.

Table S1 Amorphous TPN film Free triplets Kinetic fitting at 560nm

	τ_3(ps)	A_1 (%)	τ_{TPS}(ps)	A_2 (%)	τ_1(ps)	A_3 (%)
0GPa	443.8	13.1	9.875	29.3	1.889	57.6
0.73GPa	1386	9.1	82.87	37.7	7.236	53.2
1.43GPa	1612	8.3	78.57	43.5	10.34	48.2
2.06GPa	1855	12.5	110.4	35.6	13.07	51.9
2.34GPa	2750	12.5	102.7	36.0	11.07	51.5
3.64GPa	3948	12.9	117.2	33.2	10.68	53.9

Table S2 Amorphous TPN film Triple state bondage pairs

Kinetic fitting at 900 nm

	τ_2(ps)	A_1 (%)	τ_{ST}(ps)	A_2 (%)	τ_{TF}(ps)	A_3 (%)
0GPa	1754	32.4	227.0	37.4	17.51	30.2
0.73GPa	187	21.2	35.48	37.5	4.935	41.3
1.43GPa	93.53	32.5	14.22	42.9	1.753	24.6
2.06GPa	127.7	27.2	18.96	54.4	2.241	18.4
2.34GPa	166.9	21.4	22.82	45.5	3.198	33.1
3.64GPa	143.3	20.8	22.15	36.6	4.407	42.6

Reference

1. Grieco, C.; Doucette, G.S.; Pensack, R.D.; Payne, M.M.; Rimshaw, A.; Scholes, G.D.; Anthony, J.E.; Asbury, J.B. Dynamic Exchange During Triplet Transport in Nanocrystalline TIPS-Pentacene Films. *J. Am. Chem. Soc.* **2016**, *138*, 16069.
2. Mulazzi, E.; Ripamonti, A.; Wery, J.; Dulieu, B.; Lefrant, S. Theoretical and experimental investigation of absorption and Raman spectra of poly(paraphenylene vinylene). *Physical Review B* **1999**, *60*, 16519-16525.
3. Paudel, K.; Moghe, D.; Chandrasekhar, M.; Yu, P.; Ramasesha, S.; Scherf, U.; Guha, S. Pressure dependence of singlet and triplet excitons in amorphous polymer semiconductors. *EPL* **2013**, *104*, 27008.
